# The Histological and Biochemical Assessment of Monoiodoacetate-Induced Knee Osteoarthritis in a Rat Model Treated with Salicylic Acid-Iron Oxide Nanoparticles

**DOI:** 10.3390/biology13050331

**Published:** 2024-05-10

**Authors:** George Bică, Otilia-Constantina Rogoveanu, Florin-Liviu Gherghina, Cătălina-Gabriela Pisoschi, Sandra-Alice Buteică, Cristina-Elena Biță, Iulia-Alexandra Paliu, Ion Mîndrilă

**Affiliations:** 1 Department of Physical Medicine and Rehabilitation, University of Medicine and Pharmacy of Craiova, 2 Petru Rares Street, 200349 Craiova, Romania; bicageorgebrailoiu@gmail.com (G.B.); otilia.rogoveanu@gmail.com (O.-C.R.); gfl1972@hotmail.com (F.-L.G.); 2 Department of Biochemistry, University of Medicine and Pharmacy of Craiova, 2 Petru Rares Street, 200349 Craiova, Romania; c_pisoschi@yahoo.com; 3 Faculty of Pharmacy, University of Medicine and Pharmacy of Craiova, 2 Petru Rares Street, 200349 Craiova, Romania; 4 Department of Rheumatology, University of Medicine and Pharmacy of Craiova, 2 Petru Rares Street, 200349 Craiova, Romania; cristina.gofita@umfcv.ro; 5 Department of Pharmacology, University of Medicine and Pharmacy of Craiova, 2 Petru Rares Street, 200349 Craiova, Romania; iulia_paliu@yahoo.com; 6 Department of Anatomy, University of Medicine and Pharmacy of Craiova, 2 Petru Rares Street, 200349 Craiova, Romania; ion.mindrila@umfcv.ro

**Keywords:** monoiodoacetate, knee osteoarthritis, iron oxide nanoparticles, salicylic acid, murine model, cytokine, oxidative stress

## Abstract

**Simple Summary:**

Osteoarthritis is regarded as the most prevalent orthopedic disease affecting the elderly population worldwide. The present study investigated the effect of the orally administered salicylic acid-functionalized iron oxide nanoparticles (SaIONPs) on a murine model of knee osteoarthritis induced by monoiodoacetate. Our results proved that this novel therapeutic approach alleviated the histological dysfunction associated with this pathology suggesting the fact that nanoparticles may represent a promising alternative treatment in the early onset of knee osteoarthritis. Furthermore, our experiments proved that some biomarkers of oxidative stress and inflammatory response were improved in the group treated with SaIONPs. The findings of this pilot study suggest that SaIONPs may be a potential option in the management strategy of knee osteoarthritis. Further studies are needed to accurately adjust SaIONPs doses and to identify the mechanisms involved in generating their chondroprotective and anti-inflammatory effects.

**Abstract:**

Iron oxide nanoparticles (IONPs) represent an important advance in the field of medicine with application in both diagnostic and drug delivery domains, offering a therapeutic approach that effectively overcomes physical and biological barriers. The current study aimed to assess whether oral administration of salicylic acid-functionalized iron oxide nanoparticles (SaIONPs) may exhibit beneficial effects in alleviating histological lesions in a murine monoiodoacetate (MIA) induced knee osteoarthritis model. In order to conduct our study, 15 Wistar male rats were randomly distributed into 3 work groups: Sham (S), MIA, and NP. At the end of the experiments, all animals were sacrificed for blood, knee, and liver sampling. Our results have shown that SaIONPs reached the targeted sites and also had a chondroprotective effect represented by less severe histological lesions regarding cellularity, altered structure morphology, and proteoglycan depletion across different layers of the knee joint cartilage tissue. Moreover, SaIONPs induced a decrease in malondialdehyde (MDA) and circulating Tumor Necrosis Factor-α (TNF-α) levels. The findings of this study suggest the therapeutic potential of SaIONPs knee osteoarthritis treatment; further studies are needed to establish a correlation between the administrated dose of SaIONPs and the improvement of the morphological and biochemical parameters.

## 1. Introduction

Iron oxide nanoparticles (IONPs) represent a novel tool in the field of cosmetics, pharmaceuticals, and food sciences. IONPs are characterized by a dimension that varies between 1 to 100 nm and properties that depend on the surface functionality and size. In the medical field, IONPs exhibit uses in diagnosis and/or treatment, a fact made possible by certain specific chemical and physical proprieties, drug loading and release efficiency, and their reduced or even absent toxicity [1].

Iron oxide nanoparticles are an important member of the nanoparticle family, finding multiple uses in regenerative medicine, tissue engineering drug delivery, magnetic resonance imaging due to their biodegradability and biocompatibility etc. [2].

The main three methods used in obtaining iron oxide nanoparticles are chemical, biological, and physical. Chemical methods involve means such as co-precipitation, microemulsion, thermal decomposition, and microwave-assisted synthesis, while physical routes include laser ablation synthesis in solution and pyrolysis [3].

Bounding or loading certain pharmacologically active compounds to iron oxide nanocarriers overcomes certain difficulties, such as high toxicity, short circulating half-life, nonspecific delivery, and poor solubility of the drugs. The mechanisms involved in targeting specific sites include passive targeting, which relies on the enhanced permeability and retention of effect, or active targeting through the use of an external magnetic field [4].

Salicylic acid, which was first extracted from willow bark, has historically shown high efficiency in treating inflammatory conditions such as gout, rheumatic arthritis, rheumatic fever, and headache. Its main side effects are an unpleasant taste and gastric irritation, which were later addressed by developing a new acetyl derivative, aspirin [5].

The identification of the action mechanism made aspirin one of the most prescribed treatments for inflammatory conditions. Several studies have demonstrated aspirin efficacy in knee osteoarthritis treatment by inhibiting a secondary pathway of hyperalgesia and the reduction of cartilage loss [6,7].

The pharmacological efficacy of salicylates is primarily attributed to their capacity to inhibit cyclooxygenases (COX), consequently altering the biosynthesis of prostaglandins and thromboxane A2. The inhibition of COX activity is fundamental to the clinical efficacy of salicylates, serving as the basis for their roles as antipyretic, anti-inflammatory, and analgesic agents. Moreover, emerging evidence suggests that salicylates exert multiple cyclooxygenase-independent actions, notably through their antioxidant properties, which likely contribute to their overall therapeutic benefits. Although salicylates are recognized antioxidants due to their ability to scavenge hydroxyl radicals, the precise mechanisms underlying their antioxidant effects remain incompletely characterized [8].

Recent experiments have shown that localized and sustained delivery of a salicylic acid-based polymer provided an improved outcome of diabetic bone regeneration by significantly reducing inflammation, osteoblast activity, and density and increasing osteoblastogenesis in diabetic animals [9]. Peak plasma concentration of acetylsalicylic acid ranged from 20–30 min to 4–6 h depending on the conventional pharmaceutical formulation [10]. Through functionalizing IONPs with salicylic acid (SaIONPs), a previous study has demonstrated blood persistence of up to 24 h of the functionalized nanoparticles [11].

The liver and spleen are responsible for the main uptake of the SaIONPs. Moreover, lymph nodes, bones, heart, and lungs have also been shown to exhibit iron oxide nanoparticle accumulation [12]. Previous studies have shown that IONPs have the potential to influence bone metabolism. Furthermore, studies have demonstrated that daily administration of IONPs can alleviate osteoporosis by scavenging reactive oxygen species in murine models. IONPs can be assimilated by the cells via endocytosis, presenting a highly efficient method for targeting bone tissue in clinical applications. Furthermore, IONPs have shown promise in improving angiogenesis and facilitating the formation of new blood vessels in bone injury, both with and without the intervention of a magnetic field. Smaller IONPs with longer half-lives in the bloodstream exhibit enhanced bone-targeting properties. Additionally, IONPs can be loaded with various genes and cytokines for precise delivery at the sites of bone lesions [13,14].

Oxidative stress is the result of the imbalance between reactive oxygen species (ROS) and the body’s antioxidant defense system. ROS are highly reactive molecules or radicals produced during the incomplete reduction of oxygen or through subsequent ROS interactions [15]. This imbalance can contribute to various human diseases, including cancer and atherosclerosis, as well as accelerating the aging process itself. Therefore, mechanisms such as antioxidants play a crucial role in regulating oxidative stress and maintaining overall health [16]. Oxidative stress is increasingly recognized as a significant factor in the development of knee osteoarthritis (KOA), among other molecular mechanisms. Persistent oxidative stress exacerbates the situation by promoting the production of pro-inflammatory cytokines such as tumor necrosis factor-α (TNF-α), interleukin-1β (IL-1β), interleukin-6 (IL-6), and chemokines, triggering the release of catabolic enzymes, and ultimately contributing to the degradation of articular cartilage. This creates a feedback loop where ROS and inflammation stimulate each other’s activity. Consequently, interventions aimed at reducing oxidative stress and inflammation hold promise as potential therapeutic strategies for managing the progression of knee OA [17,18].

SaIONPs have been proven to exhibit high biocompatibility in both murine and avian models for both gavage and oral administration. Although intraperitoneal and intravenous pathways are the most studied administration routes for SaIONPs, oral administration is better tolerated by the patient and has greater convenience. [19].

Our study aimed to assess whether SaIONPs may reach the articular site and provide a beneficial effect regarding histological and biochemical parameters associated with KOA. To achieve this goal, we employed a murine model characterized by monoiodoacetate (MIA)-induced osteoarthritis for our experimental investigations. We have also investigated liver samples to confirm whether the NP was administered correctly through gavage.

## 2. Materials and Methods

### 2.1. Animals and Study Design

**Figure 1:**
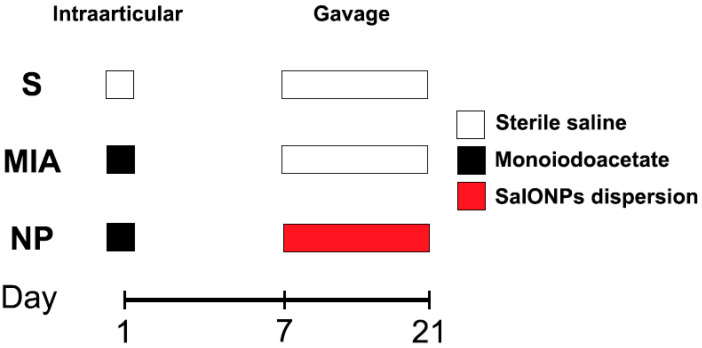
Flowchart of the experiment.

Fifteen 8-week-old Wistar male rats ranging between 300 and 350 g (average 332.5 g) were randomly assigned to 3 work groups as shown in Figure 1:

Sham (n = 5), animals that received a single 50 microliter injection with sterile saline in their right knee and were not administered any form of treatment. From day 7 to day 21, animals received 1 mL of saline;

MIA (n = 5) animals that received an injection with mono iodoacetate (Merck^®^, Darmstadt, Germany, Sodium Iodoacetate 57858-5G-F) in their right knee and were not administered any form of treatment. From day 7 to day 21, animals received 1 mL of saline;

NP (n = 5) animals that received a single dose injection with monoiodoacetate. From day 7 to day 21, animals received oral administration of SaIONP treatment.

All animals were housed individually in stainless steel ventilated cages and were provided free access to water and standard laboratory food under a 12-h light/night cycle at a constant temperature of 20 °C.

All animals were granted by the Animal Facility Unit of the University of Medicine and Pharmacy of Craiova. The experimental procedures were performed following the guidelines of the Committee of Ethics and Scientific Deontology of the University of Medicine and Pharmacy of Craiova (No.57/16.02.2023).

### 2.2. Induction of the Model and Biological Sampling

Before the experimental procedure, all animals received intraperitoneal anesthesia with a solution of ketamine (60–80 mg/kg)/xylazine (6–8 mg/kg). The fur on the right knee was removed using an electric trimmer, and the skin was disinfected using a 70% ethanol solution. Animals from MIA and NP groups were subjected to a single intraarticular shoot of 2 mg/50 µL MIA solution. After 7 days, animals from the NP group received daily administration of 1 mL of SaIONPs suspension for 14 days. Following the end of the experimental procedures, all animals were euthanized, blood samples were gathered in EDTA-coated containers and liver samples, and the right knees were harvested.

### 2.3. Chemicals

In this study, SaIONPs were synthesized via the modified Massart method, as previously described. The dispersion of SaIONPs in ultrapure water exhibited an average hydrodynamic diameter of 73 nm, a polydispersity index of 0.14, a zeta potential of +50.5 mV, a Fe_3_O_4_ concentration of 0.67 mg/mL, and an estimated salicylic acid content of 0.46 mg/mL [11].

### 2.4. Histopathological Assessment

After the animals were sacrificed, the right knees and liver samples were harvested and fixed in 10% formaldehyde for 72 h. Using a sharp microtome razor, soft tissue was removed in order to prepare the joints for decalcification in an 8% formic acid solution, which was replaced daily. After 14 days, the samples achieved the desired consistency for histological processing. Excess tissue was later removed and the knees were sectioned in a frontal plane, placed in histological cassettes, and both liver and knee samples were subjected to a standard laboratory protocol for paraffin embedding. Serial 10 µm joint and 7.5 µm liver sections were cut using a Leica RM 2235 rotary microtome. The sections were later collected on poly-L-lysine treated slides and subsequently deparaffinized in xylene and alcohol baths. The samples were stained with Hematoxylin/Eosin (Leica Biosystems^®^, Infinity 2.0) and Toluidine blue (Merk^®^, Toluidine Blue O T-3260) for general assessment and identification of the proteoglycan content in the articular cartilage. Pearls Prussian blue (Bio Optica^®^) staining was performed for identification of the SaIONPs deposits in both liver and joint specimens. All slides were coverslipped with coverslipped DPX mountant (Merck^®^, DPX Mountant for histology, 44581). Cartilage alteration was blindly assessed using a modified Mankin scale under light microscopy by two independent evaluators [20,21].

### 2.5. Biochemical Analysis

Following centrifugation at 1100× *g* for 10 min at 4 °C, the plasma and erythrocyte portions were isolated and stored at −80 °C for later analysis. All experimental measurements were conducted in triplicate and are presented as the average of three analyses ± standard deviation.

#### 2.5.1. Evaluation of Biomarkers for Oxidative and Nitrosative Stress

For the assessment of the total antioxidant capacity (TAC), malondialdehyde (MDA) catalase (CAT), and reduced glutathione (GSH), we utilized methods as previously described [21,22,23].

TAC analysis was conducted by diluting the plasma samples in 0.01 M Phosphate Buffer Solution and subsequently adding 0.1 mM 2,20-diphenyl-1-picrylhydrazyl radical reagent (DPPH). Following a 30-min incubation period in a dark environment at 20 °C, the samples were subjected to centrifugation for 3 min at 20,000× *g* and 5 °C using an Eppendorf 5417 R centrifuge. The absorbance was then measured at 520 nm using a UV-VIS spectrophotometer Kruss. Results are expressed as mmol DPPH/L.

The MDA assessment began by diluting plasma in a 1:9 volume ratio of a 1:1 mixed solution of 35% trichloroacetic acid/0.2 M Tris-Cl. and incubating the resulting mixture at 20 °C for 10 min. Subsequently, a mixture of 2 M sodium sulfate in 0.05 M thiobarbituric acid was added, followed by a second incubation at 95 °C for 45 min. After cooling on ice for 6 min, a 70% trichloroacetic acid solution was added, and the mixture was vortexed and centrifuged. The supernatant was later on assessed at 532 nm using a Kruss UV-VIS spectrophotometer. Results are expressed utilizing the molar extinction coefficient of the MDA-TBA adduct (1550 × 105 mM^−1^ cm^−1^).

To assess CAT activity, the lysate was diluted and added to a 0.07 M Phosphate Buffer Solution. Subsequently, the mixture was incubated at 37 °C for 10 min and subjected to hydrogen peroxide treatment. The evaluation of the samples was performed using a Beckman Coulter DU-65 UV-VIS spectrophotometer at 240 nm. Results are expressed as units per milligram of hemoglobin (U/mg Hb).

For the GSH assessment, the erythrocytes were lysed using cooled redistilled water and subsequently centrifuged at 4020× *g* for 10 min at 4 °C. The resulting solution was then treated with a 5% trichloroacetic acid, vortexed, and separated through centrifugation. Later on, a mixture of 0.07 M Phosphate Buffer Solution and 0.1 M Ellman’s Reagent was added to the samples, followed by incubation in a dark environment. The samples were later analyzed at 412 nm using UV-VIS spectrophotometry. GSH concentration was obtained using a standard curve. Results are presented as milligrams per deciliter (mg/dL).

Myeloperoxidase (MPO) levels were evaluated following the plasma centrifugation at 20 °C for 15 min at 1000× *g*, using a Rat Myeloperoxidase ELISA Kit (Abbexa^®^, abx513486). The sample preparation procedures followed the manufacturer’s standard protocols. The results are presented as nanograms per milliliter (ng/mL).

The nitric oxide (NO) levels were determined using a Nitric Oxide Assay Kit (Sigma^®^, MAK454-1KT). Results are reported as µM.

#### 2.5.2. Cytokine Assessment

TNF-α levels were determined using a Rat ELISA kit (Biovendor^®^, Asheville, NC, USA, RAF-130R), according to the manufacturer’s instructions. Results are reported in picograms per milliliter (pg/mL).

### 2.6. Statistical Analysis

GraphPad Prism software version 8.0.1 (GraphPad Software^®^, San Diego, CA, USA) was employed for statistical analysis. Continuous data are expressed as the mean ± standard deviation (SD). Group differences were assessed using one-way ANOVA on ranks (Kruskal–Wallis) analysis. For non-parametric data comparison, a Mann–Whitney test was applied. Statistical significance was set at *p* < 0.05.

## 3. Results

### 3.1. Histolopathological Assessment

**Figure 2:**
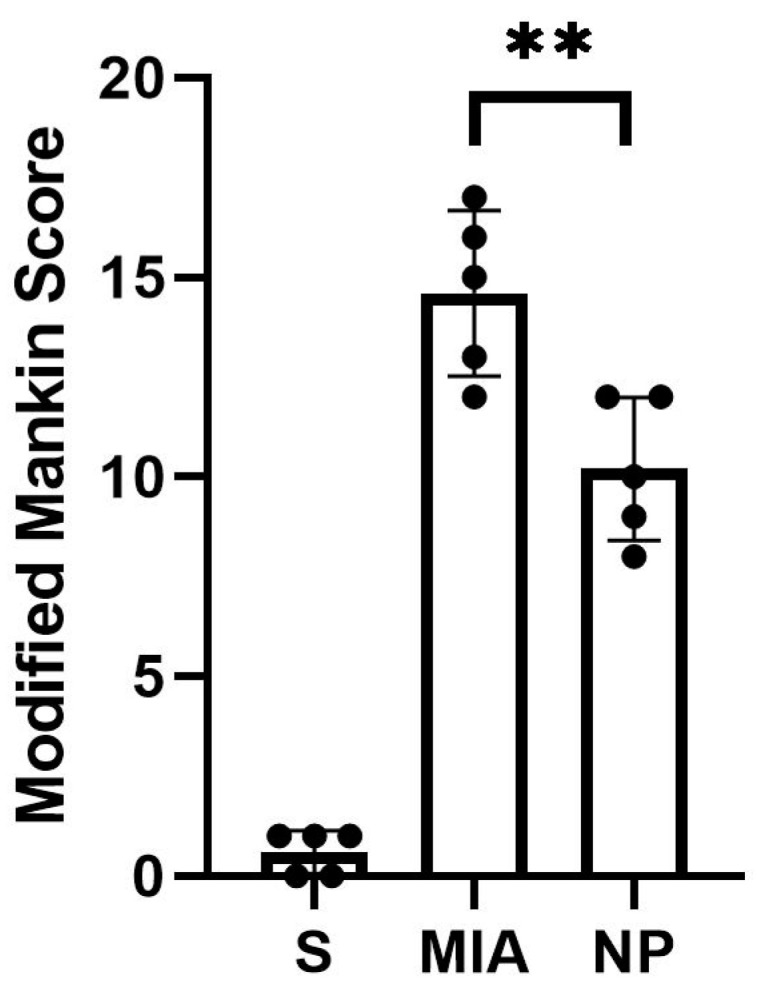
The modified Mankin score of the analyzed groups showed a significant decrease in the intraarticular lesions in the NP group compared to the MIA group (Mann–Whitney test, ** *p* < 0.01).

Animals from both NP and MIA groups experienced cartilage lesions, which were more severe in the MIA group, highlighted by an elevated modified Mankin score (Figure 2).

**Figure 3:**
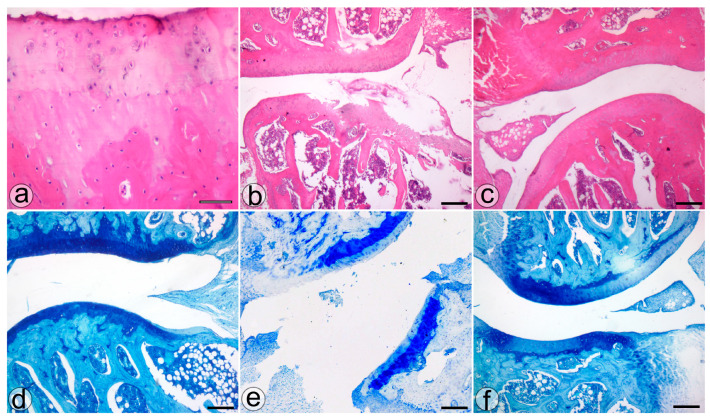
The histological features of the knee joint in the S (**a**,**d**), MIA (**b**,**e**), and NP (**c**,**f**) treated groups. H&E (**a**–**c**) and TOL (**d**–**f**) staining. Bar = 50 µm (**a**) and 200 µm (**b**–**f**).

The histological assessment highlighted more severe cartilage lesions in the MIA group. The cartilage abnormalities found in group MIA ranged from more than three fissures and cartilage loss in the superficial zone to loss of cartilaginous tissue, which extended into the deep zone, while animals assigned to group NP showed lesions ranging from cartilage loss in the superficial layer to 1 to 3 fissure that extended in the deep zone. Regarding the proteoglycan content, the MIA group exhibited lesions characterized by a decrease in the proteoglycan content in the superficial and medial layers that extended down to the deep zone. Animals from the NP group showed less severe lesions that varied from decreased content of proteoglycan in superficial and upper middle zones to a decrease in all three layers. The cellularity assessment highlighted variations ranging from hypercellularity (stage 1 Mankin) to diffuse hypocellularity (stage 3 modified Mankin) in the MIA group, while the NP group experienced only hypercellularity and clustering (stage 1 and 2 modified Mankin). Tidemark integrity was affected in both MIA and NP subjects. Apart from the items addressed by the Mankin score, we have also noticed the presence of inflammatory infiltrate in both the menisci and synovia in the MIA and NP groups (Figure 2 and Figure 3).

**Figure 4:**
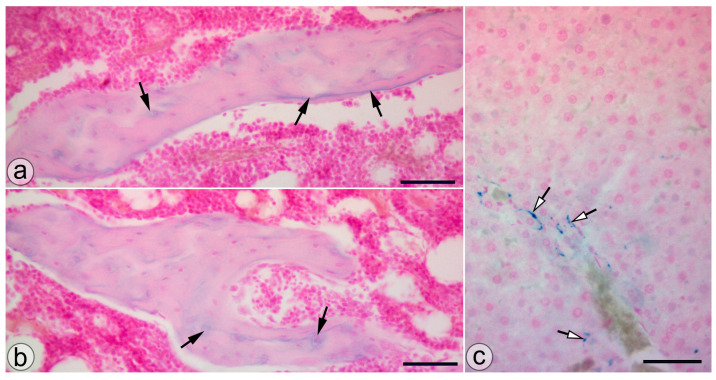
SaIONP deposits (black arrows) in the knee joint (**a**,**b**) and the periportal area (white arrows) of the liver lobule. (**c**) Pearls Prussian blue staining. Bar = 50 µm.

Animals from the NP group presented SaIONP deposits in the trabeculae of the knee subchondral bone and in the periportal areas of the liver lobules (Figure 4).

### 3.2. Biochemical Assessment

**Figure 5:**
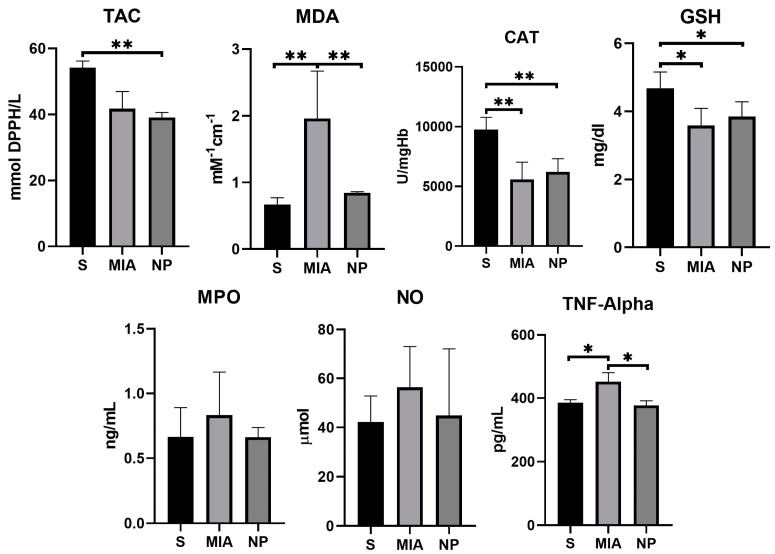
Graphic representation of the biochemical markers values investigated. (*, *p* < 0.05; **, *p* < 0.01).

MDA levels showed a significantly higher concentration in the MIA group compared to NP and S groups (*p* < 0.01), suggesting a decrease in this biomarker of oxidative stress. TNF-α activity was reduced in both S and NP groups in comparison with the MIA group (*p* < 0.01), suggesting an anti-inflammatory effect of the NP. Although NO and MPO levels were reduced in the NP group, no significant statistical correlation was observed. The TAC, GSH, and CAT activities were similar in both NP and MIA groups (as presented in Figure 5 and Table 1).

## 4. Discussion

Knee osteoarthritis (KOA) is a common rheumatic disorder in the adult population. The disease can be classified either as primary/idiopathic or secondary, linked to factors such as malposition, endocrine disorders, aseptic osteonecrosis, trauma, and metabolic disorders [7]. When the compensation mechanism that maintains the equilibrium of the cartilaginous matrix synthesis and degradation is exceeded by certain enzymatic and mechanical processes, the disease evolves from a reversible matrix degradation to an impairment in the joint congruence and, later on, in a total loss of congruence and the formation of subchondral cysts, osteophytes, and sclerosis. KOA is not only a disease that involves the hyaline cartilage but also affects the ligaments, the bony tissue, the capsule, the menisci, and the muscles that compose and mobilize the articulation [24].

Early stages of the disease are traditionally managed in a conservatory approach, which involves pharmacological and non-pharmacological means such as lifestyle changes, physical therapy, physical aids, and dietary supplements such as chondroitin, glucosamine, curcuminoids, boswelic acids, and diacereins. Minimally invasive techniques that involve intraarticular injections of certain substances that aim to improve joint homeostasis may be performed [25]. To alleviate the pain that occurs in KOA, topical application and oral administration of non-steroidal anti-inflammatory drugs (NSAIDs) were proven to be safe and efficient, especially in elderly patients over 75 years old. However, the incidence of adverse effects of systemically administered NSAIDs can limit the treatment’s efficacy and must be carefully administered with caution in patients suffering from renal impairment, cardiac disease, or who are highly susceptible to gastrointestinal bleeding, and although opioids have a limited sustained effect regarding osteoarthritic pain, concerns are raised over cognitive adverse effects and abuse potential [26,27].

A recent study has shown that the administration of low doses of acetylsalicylic acid (under 4 mg per kg body weight per day) reduced the medial plateau loss of cartilage in patients suffering from symptomatic KOA [7]. Furthermore, the same effects were observed in our study, where the dose administered was under 1.4 mg per kg bodyweight per day. This fact may be the result of the longer persistence of the SaIONPs in the bloodstream [11].

Potential chondroprotective mechanisms exhibited by the salicylic acid involve inhibiting chondrocyte apoptosis, suppressing nuclear factor kappa-light-chain-enhancer of activated B cells (NF-κB) and matrix metalloproteinase activity, as well as regulating nitric oxide synthesis [28].

One of the most challenging aspects of the non-surgical treatment of osteoarthritis is the rapid clearance and poor bio-availability of the currently used drugs. Nanoparticles represent a novel approach to overcoming these issues by acting as carriers that can enhance drug stability by incorporating the pharmacologically active compounds either on the surface or within the matrix, shielding them from enzymatic degradation. By enhancing penetration across the cartilage matrix and regulating drug pharmacokinetics through nanoparticle carriers, mitigation of the toxicity of the compounds and enhanced efficacy may be achieved. Additionally, this pharmaceutical form can be tailored to selectively target particular components or cells within the cartilage [29]. Although nanomedicine products used for diagnosis or therapy have been primarily designed for parenteral injection, predominantly administered intravenously, products intended for nasal and pulmonary delivery exist with limited application. Solid nanoparticles can undergo receptor-mediated endocytosis in the gastrointestinal tract; this process is contingent upon factors such as particle size, shape, surface properties, and the specific animal model being used [30]. Oral administration offers several advantages, including increased patient compliance and comfort, greater dosage flexibility, and a reduced risk of infection and contamination. It also ensures extensive absorption in the gastrointestinal tract. Moreover, oral administration allows for self-administration with flexible dosage options, easily customizable to meet individual patient needs, making it the preferred route of administration. Concerns surrounding oral nanoparticle delivery encompass several factors, such as limited permeation across the intestinal epithelium and the acidic pH environment present in the stomach [31]. Previous studies have shown that SaIONPs have effectively overcome these challenges and demonstrated versatility for oral administration. Their presence has been identified in the lamina propria and capillaries of the stomach, small intestine, and proximal colon, indicating their resilience to pH variations and successful absorption throughout the gastrointestinal tract [19]. The toxicity over the liver is expected to occur at much higher concentrations of cumulative doses of SaIONPs corresponding to 6 mg/kg Fe_3_O_4_. Furthermore, the salicylic acid content is also in the safe range, as doses up to 4.8 mg/kg/day of salicylates were administered orally without any observed side effects over the liver [32,33]. The presence of SaIONPs in the trabecular bone, proved by our study, confirms furthermore that oral administration of NPs constitutes a viable option for articular targeting.

Exposure of cells to reactive oxygen species triggers lipid peroxidation, leading to the rupture of cell walls and oxidation of membrane lipids in MDA, which exhibits toxic and mutagenic properties. This process, in turn, promotes pro-inflammatory responses observed in numerous chronic health conditions and environmental exposures. MDA is utilized as a biomarker to assess oxidative stress levels in a broad spectrum of diseases. Following its formation, it can be metabolized by various enzymes within the mitochondria or can interact with nucleic acids and proteins by binding covalently and causing biomolecular damage [34,35]. MDA has the chemical structure of an aldehyde and was proven to be elevated in the synovial fluid or the plasma of patients with KOA. Its main toxic effect consists of affecting the collagen’s integrity either by oxidation or by degradation [36]. A previous study has shown that salicylates have decreased the plasma concentration of MDA, probably by chelating actions over ions involved in pro-oxidant effects and, therefore, in MDA production [37,38].

TNF, a pro-inflammatory cytokine, is predominantly synthesized by monocytes/macrophages, while other cellular sources include mast cells, natural killer cells, neutrophils, osteoclasts, and fibroblasts. It is expressed initially as a transmembrane protein on cell surfaces before undergoing enzymatic cleavage to produce a soluble form, which is released into the bloodstream and detectable in plasma.

TNF plays a pivotal role in mediating inflammation and tissue destruction, particularly in conditions such as KOA. By releasing matrix metalloproteinases (MMPs) from synovial fibroblasts, TNF promotes chondrocyte apoptosis and articular cartilage degradation. Additionally, while also impairing the differentiation of mesenchymal stem cells (MSCs) into chondroblasts, TNF affects chondrogenesis [39].

NF-κB comprises a group of responsive transcription factors crucial in immune system functions. Triggered by cytokines from the TNF family, NF-κB swiftly activates genes controlling proliferation, inflammation, differentiation, and cell survival [40]. Recent studies have highlighted NFκB inhibition as a key anti-inflammatory mechanism of salicylates like aspirin, 5-amino salicylic acid (ASA), and sulfasalazine [41].

The average values of MDA and TNF-α observed in the NP group were statistically significantly decreased compared to the MIA group (*p* < 0.01). This result may be considered a consequence of the beneficial salicylate effects in KOA.

NO, a gaseous signaling molecule, plays a crucial role in both pathophysiological and physiological processes in the human organism. During inflammatory states, NO exhibits a dual role, both regulating functions and contributing to the pro-inflammatory/destructive processes. In osteoarthritic (OA) joints, elevated NO production, along with increased levels of other inflammatory mediators, is observed. Cytokines contribute to the exacerbation of destructive processes in OA cartilage by increasing the production of inducible Nitric Oxide Synthase (iNOS) production in chondrocytes, the sole cell type present in cartilage. NO, which, in turn, activates MMPs, reduces the synthesis of proteoglycans and collagen, and induces cellular death. Additionally, NO indirectly participates in increasing the production of pro-inflammatory cytokines in the affected articulation by stimulating the synthesis of TNF by synovial cells [42].

MPO is a crucial diagnostic tool for assessing oxidative and inflammatory stress. It belongs to the peroxidase subfamily and is primarily expressed in immune cells, notably neutrophils, monocytes, lymphocytes, and macrophages. MPO is stored in the cytoplasm in membrane-bound azurophilic granules and is released extracellularly upon stimulation through exocytosis or degranulation. The MPO antibacterial effects involve both an increase in the reactive nitrogen and oxygen species. While the controlled release of MPO at the site of infection is essential for its effective antimicrobial activities, uncontrolled degranulation can exacerbate inflammation and result in tissue damage, disregarding the presence of an inflammatory process. MPO has been incriminated in the pathogenesis of multiple chronic diseases such as diabetes, arthritis, liver diseases, cardiovascular diseases, cancer, and various types of tissue insults [43].

Previous studies have shown that the inhibition of iNOS and MPO by salicylates is dose-dependent, starting from 3 mM/L [44,45]. Our experiments have revealed that although non-statistical significant, the average values of NO and MPO in the NP group (that received salicylate for 14 days with a daily dose of 0.01 mM/kg body weight) were lower compared to the MIA group. These results suggest the necessity of increasing the salicylate dose to achieve better effects.

GSH, a molecule that actively reacts with and neutralizes unstable ROS, plays a critical role in maintaining cellular redox balance and defending against oxidative damage. GSH exists in two forms: oxidized (GSSG) and reduced (GSH). The balance between these forms is represented by the GSH/GSSG ratio, which is crucial for maintaining effective antioxidant effects. In OA, a reduction in glutathione (GSH) levels indicates a decline in the synthesis of type II collagen and proteoglycan in chondrocytes [46,47].

Daily administration of 35 mg of salicylates/kg/bodyweight has shown an increase in the GSH concentration [48]. In our study, it was observed that GHS concentrations were higher in the NP group as compared to the MIA group. However, the outcome did not prove to be statistically significant.

CAT, another ROS-scavenging molecule, plays a crucial role in breaking down hydrogen peroxide (H_2_O_2_), a key step in the antioxidant defense mechanisms of the organism [49]. By decreasing ROS, CAT reduces the severity of the degenerative processes and enhances the survival rate of the articular chondrocytes [36]. Although the phytochemical extract of willow bark, which contains salicylates, as well as aspirin, increases CAT activity in animal models of dyslipidemia, research conducted on our KOA model did not yield statistically significant results regarding the effect of SaIONPs on CAT [50].

TAC, also referred to as the nonenzymatic antioxidant capacity, is a biomarker used for evaluating the intrinsic antioxidant potential within the body [36]. An animal model of myocardial infarction has demonstrated that TAC is significantly higher during reperfusion in animals who received an intracoronary injection with aspirin [51]. In our study, the highest levels of TAC were achieved in the healthy animals (S group), and comparable results were seen between MIA and NP groups, indicating that SaIONPs do not have significant effects on this biomarker.

## 5. Conclusions

The main finding of the present study is that oral administration of SaIONPs has a potentially beneficial effect in alleviating histological and biochemical changes in the MIA-induced KOA murine model.

Moreover, based on our experimental findings, it was observed that certain biomarkers associated with oxidative stress, such as MDA, as well as those linked to inflammatory response, such as TNF-α, exhibited improvement in the group that received treatment with SaIONPs. These results suggest that SaIONPs hold potential as a therapeutic agent for addressing conditions characterized by oxidative stress and inflammation.

Although the preliminary results are promising, factors like treatment regimens and dosage still need to be determined to achieve optimal results. SaIONPs may represent a novel complementary solution in the early management of osteoarthritis and may exhibit additive effects compared to the standalone traditional therapies.

The limitations of the present work are represented by the absence of diverse comparative groups, multiple biomarker evaluations, and immunochemistry assessments, which will be addressed in upcoming studies.

## Figures and Tables

**Table 1:** Standard deviation and mean values of the biochemical markers (SD = standard deviation).

	S		MIA		NP	
	Mean	±SD	Mean	±SD	Mean	±SD
**TAC [mMol DPPH/L]**	54.18	2.06	41.75	5.24	39.13	1.47
**MDA [mM^−1^cm^−1^]**	0.67	0.10	1.96	0.71	0.85	0.02
**CAT [U/mgHb]**	9748.78	1037.35	5579.68	1452.29	6204.92	1112.12
**GSH [mg/dL]**	4.68	0.48	3.59	0.50	3.85	0.43
**MPO [ng/mL]**	0.66	0.23	0.83	0.33	0.66	0.07
**NO [µMol]**	42.24	10.60	56.36	16.67	44.80	27.30
**TNF-alpha [pg/mL]**	385.20	10.26	452.20	28.52	377.40	14.55

## Data Availability

All data are present in the main text.
